# Severe Diltiazem Poisoning Treated with Hyperinsulinaemia-Euglycaemia and Lipid Emulsion

**DOI:** 10.1155/2013/138959

**Published:** 2013-05-20

**Authors:** Nadine Monteiro, Joana Silvestre, João Gonçalves-Pereira, Camila Tapadinhas, Vitor Mendes, Pedro Póvoa

**Affiliations:** ^1^Polyvalent Intensive Care Unit, São Francisco Xavier Hospital, West Lisbon Hospital Centre, 1449-005 Lisbon, Portugal; ^2^Faculty of Medical Sciences, New University of Lisbon, 1169-056 Lisbon, Portugal

## Abstract

*Introduction*. Calcium channel blockers (CCBs) drugs are widely used in the treatment of cardiovascular diseases. CCB poisoning is associated with significant cardiovascular toxicity and is potentially fatal. Currently, there is no specific antidote and the treatment of CCB poisoning is supportive; however, this supportive therapy is often insufficient. We present a clinical case of severe diltiazem poisoning and the therapeutic approaches that were used. *Case Report*. A 55-year-old male was admitted to the intensive care unit (ICU) after voluntary multiple drug intake, including extended release diltiazem (7200 mg). The patient developed symptoms of refractory shock to conventional therapy and required mechanical ventilation, a temporary pacemaker, and renal replacement therapy. Approximately 17 hours after drug intake, hyperinsulinaemia-euglycaemia with lipid emulsion therapy was initiated, followed by progressive haemodynamic recovery within approximately 30 minutes. The toxicological serum analysis 12 h after drug ingestion revealed a diltiazem serum level of 4778 ng/mL (therapeutic level: 40–200 ng/mL). *Conclusions*. This case report supports the therapeutic efficacy of hyperinsulinaemia-euglycaemia and lipid emulsion in the treatment of severe diltiazem poisoning.

## 1. Introduction

Diltiazem is a nondihydropyridine L-type calcium channel blocker (CCB) which is widely used in the treatment of cardiovascular diseases. The prescription of CCB has increased significantly in recent years, [1, 2] and concomitantly the number of cases of voluntary and involuntary poisoning.

In 2011, the *American Association of Poison Control Centers* reported 1995 deaths from exposure to toxic substances, 1689 of which were from medications (84.7%) [3]. Following analgesics and antidepressants, cardiovascular drugs were the most often involved. Of these drugs, CCB were most commonly used [3]. 

Calcium channel blockers overdose can cause life-threatening effects, such as bradycardia, atrioventricular (AV) block, hypotension, metabolic acidosis, and shock that is often refractory to conventional therapy.

The treatment of CCB poisoning has been limited to organ support measures. The importance of hyperinsulinaemia-euglycaemia and lipid emulsion therapy has recently been recognised in the treatment of these patients [4–12]. Traditionally, these approaches are used as late salvage therapy in cases of CCB poisoning when other measures have failed.

We hereby present a clinical case of a patient with severe diltiazem poisoning in which hyperinsulinaemia-euglycaemia and lipid emulsion therapy contributed to haemodynamic stabilisation. 

## 2. Clinical Case

A 55-year-old male was admitted to the emergency room (ER) with a low level of consciousness approximately two hours after voluntary multiple drug ingestion, including diltiazem (7200 mg), perindopril (150 mg), simvastatin (280 mg), and escitalopram (600 mg).

The patient presented a past history of essential arterial hypertension, ischemic cardiac disease, dyslipidaemia, and major depression.

On the hospital admission, he was comatose with a Glasgow Coma Score (GCS) of 8, a blood pressure (BP) of 77/44 mmHg, and a heart rate (HR) of 48 bpm.

The patient began treatment with intravenous fluid, atropine, glucagon, sodium bicarbonate, and calcium gluconate. Severe bradycardia and hypotension persisted, for which a dopamine infusion was initiated (maximum dose: 7.5 mcg/kg/min).

Laboratory evaluation data are presented in Table 1. Arterial blood gases (FiO_2_ 0.3) revealed a metabolic acidosis (pH 7.306, PCO_2_ 34.0 mmHg, PO_2_ 90.3 mmHg, HCO_3_ 17.6 mmol/L, and lactate 3.9 mmol/L).

An electrocardiogram was performed and showed a sinus rhythm with 48 bpm; a first-degree atrioventricular block; and the pattern of a complete right bundle branch block.

The condition progressed to refractory shock, and the patient developed an acute renal failure with oliguria. At that time he was admitted in the intensive care unit (ICU).

Aggressive fluid resuscitation and vasopressor support with dopamine, norepinephrine, epinephrine, terlipressin, and dobutamine were initiated with poor hemodynamic response. Progressive worsening of lactic acidosis was observed (Table 2). An intravenous (iv) calcium infusion of 2 g/h was instituted, and renal replacement therapy was initiated (continuous veno-venous hemodiafiltration at effluent rates of 35 mL/Kg/h) without significant change in his cardiovascular failure.

The patient's level of consciousness deteriorated even further (GCS: 5), and he was subsequently tracheally intubated, and invasive mechanical ventilation was initiated. The patient evolved with a severe bradycardia, needing a temporary pacemaker.

After approximately 9 hours in the ICU, refractory hypotension persisted. At that time, a 20% lipid emulsion infusion was prescribed at 0.5 mL/Kg/h rate, plus (iv) short-acting human insulin at increasing doses reaching a maximum dose of 45 U/h and 30% dextrose in water infusion adjusting infusion rate for the maintenance of euglycaemia.

Approximately 30 min after the initiation of these supportive measures, progressive haemodynamic improvement was observed with the normalisation of lactic acidaemia (Table 2), which allowed a gradual weaning of vasopressor support as well as in the recovery of own cardiac rhythm.

After 48 hours in ICU, the patient was successfully weaned from ventilatory and vasopressor support.

Despite diuresis recovery, the need for renal replacement therapy persisted until haemodynamic stabilisation was observed. Continuous veno-venous haemodiafiltration was stopped and deescalated to intermittent hemodialysis. The patient was discharged from ICU on the 4th day of hospital stay. The patient experienced full recovery of renal function and returned home after 3 days.

The plasma concentration of diltiazem 12 h after ingestion was 4778 ng/mL (therapeutic level: 40–200 ng/mL), that is to say almost 24 times above the upper limit of the therapeutic range.

## 3. Discussion

In this clinical case, the authors describe a severe CCB poisoning (near 24  ×  the upper volume of the therapeutic range) that results in a severe refractory shock with multiple organ failure that only recovered with the hyperinsulinaemia-euglycaemia and lipid emulsion treatment. 

The cardiovascular effects of CCB poisoning involve the excessive blockage of the L-type calcium channels in the myocardial cell membranes of the cardiac electrical conduction system and the vascular smooth muscle tissue, thereby preventing the entry of calcium into the cells. Therefore, cardiac inotropism, dromotropism, and chronotropism are reduced along with vascular tone. In addition, CCB inhibits the influx of calcium into pancreatic beta cells and peripheral tissue which leads to the decreased excretion of insulin and peripheral resistance to the action of CCB [7, 13, 14]. Conventional therapy for CCB poisoning includes the administration of fluids, calcium salts, glucagon, and vasopressors [5, 6, 15–17].

Extracorporeal elimination by conventional haemofiltration and dialysis is not recommended because these agents bind to plasma proteins and have a large volume of distribution. The molecular adsorbent recirculating system (MARS) was successfully used in one case report of severe diltiazem poisoning [18]. However, this technique is expensive and is not always available in a timely manner.

In our case, there was rapid deterioration of the clinical condition and refractoriness to conventional therapy which was rapidly reversed after the prescription of hyperinsulinaemia-euglycaemia and lipid infusion therapy.

In normal circumstances, myocardial cells use the oxidation of free fatty acids as an energy substrate for aerobic metabolism. In CCB poisoning, the uptake of free fatty acids by the myocardium is decreased, and the myocardium uses glucose as an energy substrate. However, the decrease in tissue perfusion secondary to the excessive blockage of vascular calcium channels complicates the distribution of glucose into the tissue. Simultaneously, hypoinsulinaemia and insulin resistance prevent the uptake of glucose by myocardial cells and vascular smooth muscle, thus limiting the use of glucose as an energy substrate. The lack of energy substrate exacerbates cardiovascular depression which is already compromised by the blocking of the calcium channels.

These mechanisms led to the hypothesis that the administration of high-dose insulin to treat CCB poisoning could compensate for hypoinsulinaemia and insulin resistance and as a result could interrupt the vicious cycle that is responsible for progressive haemodynamic deterioration and, ultimately, patient death. The efficacy and safety of this treatment have been demonstrated in several cases of CCB poisoning [6–8, 10, 12]. 

Therapeutic hyperinsulinaemia-euglycaemia consists of a continuous infusion of high-dose regular short-acting insulin (0.5–1 UI/kg) with concomitant glucose infusion that is titrated to maintain glycaemia within normal limits, which may necessitate a glucose dosage of 15–30 g/h.

Lipid emulsion has been used to treat poisoning by local anaesthetics. There is insufficient data to support the use of lipid emulsion as a first-line option; however, this therapy has been used as salvage therapy in pharmaceutical poisoning by other lipophilic drugs, particularly CCBs [9, 12]. The exact mechanism of action of this treatment is not known. The most widely accepted theory is that the emulsion acts as a “lipid sink,” surrounding a lipophilic drug molecule and rendering it ineffective. 

## 4. Conclusions

The combination of hyperinsulinaemia-euglycaemia and lipid emulsion therapy was effective for the haemodynamic recovery of a patient with refractory cardiogenic shock secondary to severe diltiazem poisoning. The early prescription of these therapies in patients with CCB poisoning may improve their prognosis. 

## Figures and Tables

**Table 1:** Laboratory data.

	Hospital admission	UCI admission	12 hrs after ICU admission	48 hrs after ICU admission
Red blood cells (×10^12^/L)	4.72	3.9	3.94	4.01
Haemoglobin (g/dL)	14.0	11.5	11.7	12.1
White cell count (×10^9^/L)	10.5	23.8	19.0	19.1
Platelet count (×10^9^/L)	224	216	127	93
Prothrombin time (sec)	10.9	11.8	13.7	11.0
C-reactive protein (mg/dL)	0.41	0.49	11.3	9.8
Urea (mg/dL)	40	49	50	23
Creatinine (mg/dL)	1.22	1.79	2.47	1.70
Glucose (mg/dL)	225	306	275	186
Potassium (mmol/L)	3.56	4.54	3.85	4.2
Sodium (mmol/L)	135	137	137	135
Calcium (mg/dL)	8.5	6.6	9.7	8.8
Alanine transaminase (U/L)	36	49		
Aspartate transaminase (U/L)		28		
*γ*-glutamyltransferase (U/L)	55	119		
Total bilirubin (mg/dL)	0.37	0.88	0.44	0.46

**Table 2:** Evolution of arterial blood gases in the ICU.

	0 h (FiO_2_: 0.3)	4 h* (FiO_2_: 0.6)	9 h (FiO_2_: 0.8)	10 h (FiO_2_: 0.6)	12 h (FiO_2_: 0.5)	48 h**(FiO_2_: 0.4)
pH	7.241	7.242	7.195	7.263	7.35	7.396
PaCO_2_ (mmHg)	46.1	35.5	29.3	31.1	31.4	38.8
PaO_2_ (mmHg)	68.6	72.5	207	188	217	134
HCO^−3^ (mEq/L)	16.7	15.5	12.6	15.1	18.8	23.8
Lactate (mmol/L)	4.3	5.7	9.4	6.7	3.4	0.8

*Intubated and invasive mechanical ventilation was initiated.

**Extubated.
